# Preparation of ^177^Lu-PSMA-617 in Hospital Radiopharmacy: Convenient Formulation of a Clinical Dose Using a Single-Vial Freeze-Dried PSMA-617 Kit Developed In-House

**DOI:** 10.1155/2021/1555712

**Published:** 2021-11-20

**Authors:** Mohini Guleria, Jeyachitra Amirdhanayagam, Haladhar D. Sarma, Ramya Priya Rallapeta, V. S. Krishnamohan, Ajit Nimmagadda, Parthasarathy Ravi, Sailaja Patri, Tekchand Kalawat, Tapas Das

**Affiliations:** ^1^Radiopharmaceuticals Division, Bhabha Atomic Research Centre, Trombay, Mumbai 400085, India; ^2^Radiation Biology and Health Sciences Division, Bhabha Atomic Research Centre, Trombay, Mumbai 400085, India; ^3^Department of Nuclear Medicine, Sri Venkateswara Institute of Medical Sciences, Tirupati, 517507 Andhra Pradesh, India; ^4^Homi Bhabha National Institute, Anushaktinagar, Mumbai 400094, India

## Abstract

**Objective:**

In the recent time, endoradionuclide therapy for metastatic castration-resistant prostate carcinoma employing ^177^Lu-PSMA-617 has yielded encouraging results and several clinical trials with the agent are currently ongoing. Routine preparation of ^177^Lu-PSMA-617 patient doses can be made simpler and convenient, if the ingredients essential for radiolabeling are made available in a ready-to-use lyophilized form.

**Methods:**

PSMA-617 freeze-dried kit was formulated and used for the preparation of ^177^Lu-PSMA-617 clinical dose with high radiochemical purity using low/medium specific activity ^177^Lu. Detailed radiochemical studies were performed to determine the maximum activity and volume of ^177^LuCl_3_, which can be added in the kit for the formulation of ^177^Lu-PSMA-617. Studies were also performed to determine the shelf life of the kit to ensure its long-term usage. Studies were performed in buffer as well as human serum medium to determine the stability of the ^177^Lu-PSMA-617 complex after storing in respective media up to 7 days postpreparation. About ten patient doses of ^177^Lu-PSMA-617 were administered, and posttherapy scans were acquired.

**Results:**

The formulated freeze-dried kit of PSMA-617 could be radiolabeled with an average percentage radiochemical purity > 98.53 ± 0.38. The freeze-dried kit was found suitable for tolerating up to 0.5 mL of ^177^LuCl_3_ (in 0.01 N HCl) and specific activity of 555 MBq/*μ*g (15 mCi/*μ*g) for the preparation of the patient dose of ^177^Lu-PSMA-617. The ^177^Lu-PSMA-617 complex prepared using the freeze-dried kit of PSMA-617 was observed to maintain % radiochemical purity (RCP) of 96.74 ± 0.87 and 94.81 ± 2.66, respectively, even after storing up to 7 days in buffer and human serum, respectively. ^177^Lu-PSMA-617 prepared using the in-house formulated freeze-dried kit of PSMA-617 exhibited accumulation in metastatic lesions picked up in a pretherapy PET scan. Reduction in number as well as size of lesions was observed in posttherapy scans acquired after two months of administering the first therapeutic dose of ^177^Lu-PSMA-617.

**Conclusions:**

The freeze-dried kit of PSMA-617 could be used for the preparation of ^177^Lu-PSMA-617 with high radiochemical purity (>98%) in a reproducible manner. ^177^Lu-PSMA-617 prepared using the developed kit was successfully evaluated in patients suffering from metastatic prostate cancer.

## 1. Introduction

Prostate cancer is the second most frequently diagnosed cancer in men and the fifth leading cause of cancer-related deaths worldwide [1, 2]. According to the GLOBOCAN 2018 estimate, more than 1.3 million patients were expected to be diagnosed with prostate cancer leading to 359,000 associated deaths worldwide during 2018 [3]. Therefore, early screening and diagnosis followed by treatment employing a proper therapeutic regimen are essential in order to reduce the mortality rate of prostate cancer patients. Recent success of PSMA (Prostate-Specific Membrane Antigen) ligands labeled with PET (Positron Emission Tomography) radionuclides, particularly with ^68^Ga, has increased the application of nuclear imaging in the diagnosis of prostate cancer in different clinical settings, such as for primary staging, assessment of therapeutic responses, and treatment planning [4–9]. Clinical accomplishments achieved with ^68^Ga-PSMA-11 have encouraged extending the application of this ligand for radiotherapy of prostate cancer. In the recent years, success of the ^177^Lu-labeled PSMA ligand, namely, ^177^Lu-PSMA-617, has opened up a new chapter in the clinical management of prostate cancer, where well-established therapeutic efficacy of ^177^Lu in combination with targeting efficacy of the PSMA-617 ligand yielded encouraging outcomes [10–20].

Clinical efficacy of ^177^Lu-PSMA-617 reported in the contemporary literature has resulted into a steady increase in demand of ^177^Lu-PSMA-617 radiotherapy in the nuclear medicine centers. Requirement of high activity per dose administration (~3.70-7.40 GBq (100-200 mCi)) and multiple dose preparations to cater for the need of the patients require handling of considerable amount of radioactivity by hospital radiopharmacy personnel at a time [21]. Apart from radiation hazard, preparation of radiotherapeutic agents following the wet chemistry protocol at hospital radiopharmacy set-up has its own challenges, such as requirement of additional time for weighing the chemicals, preparation of buffers and other solutions, pH adjustment of the radioactive formulation, incubation of the highly radioactive preparation at an elevated temperature along with the possibility of batch failure, and batch to batch variation [22]. Development of suitable lyophilized kits may solve the majority of the above-mentioned problems, and patient doses can be formulated in a comparatively easy and convenient manner at the hospital radiopharmacy. By working in this direction, attempts were made to prepare a freeze-dried kit of PSMA-617, suitable for the formulation of the ^177^Lu-PSMA-617 patient dose using low/moderate specific activity ^177^Lu, available from a medium neutron flux research reactor employing the ^176^Lu(n,*γ*)^177^Lu route.

Herein, we report the formulation of a single-vial freeze-dried PSMA-617 kit, preparation of patient doses of ^177^Lu-PSMA-617 using the kit, detailed radiochemical studies with respect to the formulation of ^177^Lu-PSMA-617 using the lyophilized kit, pharmacokinetic evaluation of ^177^Lu-PSMA-617 in a healthy small animal model, and administration of the radiolabeled preparation in patients suffering from metastatic prostate cancer.

## 2. Experiment

### 2.1. Materials and Methods

PSMA-617 was procured from ABX advanced biochemical compounds (Germany). HEPES [(4-(2-hydroxyethyl)-1-piperazineethanesulfonic acid)], gentisic acid (2,5-dihydroxy benzoic acid), and sodium hydroxide were procured from Sigma-Aldrich (USA). Nonradioactive lutetium chloride (99.99% chemically pure) was procured from Sigma-Aldrich for its use as a carrier during radiolabeling procedures. ^177^LuCl_3_ utilized in the present experiment was produced in a medium neutron flux research reactor of Bhabha Atomic Research Centre (BARC) via ^176^Lu(n,*γ*)^177^Lu nuclear reaction and radiochemically processed following the procedure reported elsewhere [23, 24]. ^177^LuCl_3_, used during the present study, was having a specific activity in the range of 740-925 MBq/*μ*g (20-25 mCi/*μ*g) and radioactive concentration of 37 MBq/*μ*L (1 mCi/*μ*L). Various other chemicals used in the present work were of Analytical Reagent (AR) grade and procured from local manufacturers of repute.

Freeze-drying was done using the Christ Alpha 1-2 LDplus freeze-drier (Germany). Pharmaceutical borosilicate glass vials (10 mL capacity, crimp neck) and slotted rubber closures used in the present study were procured from reputed local manufacturers. The glass vials and rubber closures were thoroughly cleaned and autoclaved following the usual procedure prior to use. pH strips (nonbleeding, 0-14) used for the determination of pH of the reaction mixtures were procured from Merck (USA). A water bath (RSB-12) was procured from Remi Elektrotechnik Limited (India). Whatman (UK) 3 MM chromatography paper was used for the paper chromatography (PC) studies. High-performance liquid chromatography (HPLC) studies were carried out using a Jasco PU-2080 plus (Japan) dual pump HPLC system employing a C-18 reverse-phase HiQSil (250 × 4 mm) column. The elution profile was monitored by detecting the radioactivity signal using a Gina Star radiometric NaI(Tl) detector (Raytest, Germany) coupled with the HPLC system. All solvents used for the HPLC analyses were of HPLC grade and filtered prior to use. Durapore® Polyvinylidene Fluoride (PVDF) membrane filters (47 mm, 0.22 *μ*m), used for the filtration of HPLC solvents, were procured from Merck (India).

Radioactive counting as well as studies pertaining to animal experimentations were performed as per the procedures reported by Guleria et al. [25]. In brief, all radioactive countings were performed using a well-type NaI(Tl) scintillation detector, procured from Electronics Corporation of India Limited (India), unless mentioned otherwise. The baseline was adjusted to 150 keV and window at 100 keV, so as to utilize the 208 keV gamma photon emission of ^177^Lu during radioactive countings. An isotope dose calibrator used for the measurement of activity associated with the patient dose formulation was procured from Capintec (CRC-15 BETA, CII-Capintec, USA).

Animal experimentation was carried out in healthy male Wistar rats which were bred and reared in the laboratory animal facility of Radiation Biology and Health Sciences Division of our institute (BARC) under standard management practice. Radioactive counting associated with the biodistribution studies was carried out using a flat-type NaI(Tl) scintillation counter, procured from Electronics Corporation of India Limited, using the same set-up for baseline and window as mentioned above. The animal studies reported in the present article were approved by the competent authority, namely, Institutional Animal Ethics Committee (IAEC) of our institute (BARC). All animal experiments reported in the present article were carried out in strict compliance with the institutional (IAEC-BARC) guidelines following the relevant national laws related to the conduct of animal experimentation (Prevention of Cruelty to Animals Act, 1960).

Clinical studies were performed in male patients suffering from metastatic prostate cancer. All the patients selected for the present study had biopsy-proven metastatic prostate cancer and had undergone ^18^F-PSMA-1007 PET-CT (Computed Tomography) scans to ensure PSMA expression in the cancerous lesions 2-3 weeks prior to undergoing ^177^Lu-PSMA-617 therapy. Approval for clinical studies was obtained from the Institutional Medical Ethics Committee of the hospital (Sri Venkateswara Institute of Medical Sciences (SVIMS)), and informed written consent was obtained from each patient prior to the administration of the radiotherapeutic agent. Posttherapy scintigraphy was performed on a dual-head gamma camera (Symbia E, Siemens) equipped with a Low-Energy High-Resolution (LEHR) collimator. Anterior and posterior whole-body sweep views were performed using the 256 × 1024 matrix at the speed of 10 cms/minute.

### 2.2. Formulation of Freeze-Dried PSMA-617 Kits

Three batches of freeze-dried PSMA-617 kits, comprising of 20 kit vials per batch, were prepared under aseptic conditions following the procedure mentioned below. Stock solutions of HEPES buffer, gentisic acid, and NaOH were prepared by dissolving 2.38 g of HEPES, 1.0 g of gentisic acid, and 680 mg of NaOH separately in 8.0 mL, 4.0 mL, and 8.0 mL of HPLC grade water, respectively. A stock solution of PSMA-617 was also prepared by dissolving 2 mg of the agent in 1.0 mL of HPLC grade water. For the formulation of PSMA-617 kits, aliquots were withdrawn from the stock solutions of HEPES (1.25 M, 1.48 g, 5.0 mL), gentisic acid (0.5 g, 2.0 mL), and NaOH (170 mg, 2.0 mL) and added into a freshly autoclaved glass vial followed by the addition of 1.0 mL of PSMA-617 (2.0 mg) solution. The resulting solution (10.0 mL) was thoroughly mixed and subsequently subjected to sterile filtration using 0.22 *μ*m filters before dispensing into individual glass vials. About 0.5 mL of the sterile solution was dispensed in each glass vial. All the glass vials were closed using autoclaved slotted rubber closures and were kept for freezing overnight, first at 0°C for 4 h followed by -38°C for 12 h. Frozen content of the glass vials was then subjected to lyophilization for ~8 h, which resulted in the formation of a dried pellet. The kit vials, thus prepared, were immediately stored at 2-8°C till further use.

### 2.3. Preparation of ^177^Lu-PSMA-617 Using Freeze-Dried PSMA-617 Kits

For the preparation of ^177^Lu-PSMA-617 using the PSMA-617 kit, the lyophilized kit vial was allowed to attain the ambient temperature and subsequently reconstituted using 1.0 mL of normal saline (0.9% *w*/*v* NaCl in water). The lyophilized powder was dissolved readily forming a clear colorless solution. ^177^LuCl_3_ solution was added in the kit vial, and the resulting solution was incubated in a water bath maintained at 95°C for a period of 25 min, which resulted in the formulation of the ^177^Lu-PSMA-617 complex with high radiochemical purity.

### 2.4. Quality Control of ^177^Lu-PSMA-617

Quality control studies involving the determination of radiochemical purity of ^177^Lu-PSMA-617 were carried out by PC and HPLC. PC was carried out following the procedure reported elsewhere using acetonitrile : water (1 : 1 *v*/*v*) as the mobile phase [26]. On the other hand, reverse-phase HPLC was performed using a gradient solvent system comprising 0.1% tri-fluoro acetic acid (TFA) in water (A) and 0.1% TFA in acetonitrile (B) as the mobile phase (0-4 min 95% A, 4-15 min 95% A to 5% A, 15-20 min 5% A, 20-25 min 5% A to 95% A, and 25-30 min 95% A). The flow rate of the eluting solvent mixture was maintained at 1 mL/min, and the elution profile was monitored by detecting the radioactivity signal associated with the eluting solvent.

### 2.5. Radiochemical Studies

Detailed radiochemical studies were performed using the freeze-dried kits in order to determine the maximum amount of ^177^Lu activity and maximum volume of ^177^LuCl_3_ solution, which can be added into each kit vial without compromising the radiochemical purity of the ^177^Lu-PSMA-617 formulation.

#### 2.5.1. Determination of Maximum Activity Which Can Be Added in Each Kit Vial

A stock solution of carrier/nonradioactive lutetium (1 *μ*g/5 *μ*L) was prepared by dissolving nonradioactive LuCl_3_ in Milli-Q water. For determining the maximum ^177^Lu activity which can be added into each kit vial without compromising the radiochemical purity of ^177^Lu-PSMA-617, the lyophilized powder (PSMA-617, 100 *μ*g, 95.9 nmol) of each kit vial was dissolved in 1.0 mL of saline and a fixed amount of ^177^LuCl_3_ (20 mCi, 740 MBq, 1 *μ*g of Lu) was added in each vial along with gradually increasing volume of carrier lutetium solution in the different kit vials. Four different concentrations of carrier lutetium comprising 7.41 *μ*g (42 *μ*L), 5.73 *μ*g (34 *μ*L), 4.61 *μ*g (28 *μ*L), and 3.20 *μ*g (21 *μ*L) of Lu, which correspond to 2 : 1, 2.5 : 1, 3 : 1, and 4 : 1 molar ratios of PSMA-617 and lutetium, respectively, were used for the present study. The reaction mixtures were subsequently incubated in a water bath maintained at 95°C for 25 min. Postincubation, reaction mixtures were allowed to attain room temperature, and radiochemical purity of each reaction mixture was determined using the quality control procedures mentioned above.

#### 2.5.2. Determination of Maximum Volume of ^177^LuCl_3_ Which Can Be Added in Each Kit Vial

Experiments were also carried out in order to determine the maximum volume of ^177^LuCl_3_, which can be added in each kit vial without compromising the radiochemical purity of the ^177^Lu-PSMA-617 formulation. For this, a stock solution of ^177^LuCl_3_ was prepared with a radioactive concentration of 3.70 MBq/*μ*L (0.1 mCi/*μ*L) by diluting the original ^177^LuCl_3_ solution using 0.01 N HCl. Kits were reconstituted with normal saline (500-970 *μ*L), and gradually increasing volume (30 to 500 *μ*L) of ^177^LuCl_3_ was added in the vials. The pH of the reaction mixtures was determined using the pH strips prior to incubation. The kit vials were incubated at 95°C for 25 min, and the percentage radiochemical purity of each reaction mixture was determined by the quality control procedures mentioned earlier.

### 2.6. Preparation of the ^177^Lu-PSMA-617 Patient Dose Using the Freeze-Dried Kit of PSMA-617

For the preparation of the patient dose of ^177^Lu-PSMA-617, the freeze-dried kit was first allowed to attain ambient temperature and subsequently reconstituted using 1.0 mL of saline. Required volume of ^177^LuCl_3_ (3.70-5.55 GBq (100-150 mCi), 100-150 *μ*L) was added in the kit vial, and the reaction mixture was incubated at 95°C in a water bath for 25 min. Postincubation, the reaction mixture was allowed to attain the room temperature. An aliquot (~10 *μ*L) was withdrawn from the reaction mixture for quality control studies, which were performed following the procedures mentioned above. The rest of the preparation was subjected to filtration using sterile Millipore filters (0.22 *μ*m). The activity, postfiltration, was measured using an isotope dose calibrator to determine the loss of activity incurred during the formulation and filtration process. Finally, the sterile ^177^Lu-PSMA-617 preparation was administered in patients, subject to the clearance of all quality control parameters.

### 2.7. *In Vitro* Stability of ^177^Lu-PSMA-617 Formulated Using the Freeze-Dried Kit

To determine the *in vitro* stability of the ^177^Lu-PSMA-617 complex prepared using the in-house formulated freeze-dried PSMA-617 kit, radiolabeled preparation was stored at room temperature till 7 d postpreparation and the radiochemical purity of the preparation was determined at two different postpreparation time points viz. 1 d and 7 d by employing the quality control techniques mentioned earlier.

### 2.8. Stability of ^177^Lu-PSMA-617, Formulated Using the Freeze-Dried Kit of PSMA-617, in Human Serum

The stability of ^177^Lu-PSMA-617, formulated using the freeze-dried kit, in human blood serum was evaluated by adding an aliquot (100 *μ*L) of the radiolabeled preparation to freshly isolated human blood serum (500 *μ*L) and incubating the preparation at room temperature up to 7 days postpreparation. A small aliquot (50 *μ*L) was withdrawn from the mixture at desired time points viz. 1 d, 2 d, 5 d, and 7 d postpreparation, and 450 *μ*L of acetonitrile was added to the withdrawn solution in order to precipitate the serum proteins. The mixture was centrifuged at 10000 rpm for 10 minutes, and the supernatant solution was carefully separated from the precipitate. Finally, a small aliquot of the supernatant was analyzed by HPLC following the procedure mentioned above.

### 2.9. Shelf Life of the Freeze-Dried PSMA-617 Kit

For determining the shelf life of the freeze-dried kits, kit vials were stored at 4°C up to a period of one year. Kit vials were randomly selected after 3 months, 6 months, and one year of storage and radiolabeled using ^177^LuCl_3_ following the protocol mentioned earlier. The percentage radiochemical purity of ^177^Lu-PSMA-617 was determined by the aforementioned quality control techniques.

### 2.10. Biodistribution Studies

Bioevaluation of ^177^Lu-PSMA-617, prepared using the freeze-dried PSMA-617 kit, was carried out in healthy male Wistar rats at four different postadministration time points viz. 3 h, 1 d, 2 d, and 7 d by following the procedures reported elsewhere by Guleria et al. [25]. The radiolabeled formulation was diluted using sterile saline prior to administration. One of lateral tail veins was utilized for administering an aliquot of the diluted solution (100-150 *μ*L, ~3.70-5.55 MBq (100-150 *μ*Ci)) in each animal. Postinjection all the animals were kept at the normal laboratory atmosphere with adequate supply of food and water. Carbon dioxide asphyxia was used for sacrificing the animals at the different postadministration time points (3 h, 1 d, 2 d, and 7 d) using three animals for each time point. Blood samples were collected from each animal by cardiac puncture immediately after sacrificing the animals. Following that, dissection of the animals was carried out and various organs were excised and weighed using a weighing balance. Determination of activity associated with various organs/tissues was carried out by counting the respective organ/tissue in a flat-type NaI(Tl) counter. Data obtained from aforementioned studies was utilized for calculation of the percentage of injected activity (%IA) accumulated in various organs/tissues and expressed as %IA per organ/tissue [25]. Calculation of the total activity accumulated in the blood, skeleton, and muscles was carried out by considering 7%, 10%, and 40% of the animal body weight being constituted by these organs/tissues, respectively [25, 27, 28]. The activity excreted via urinary excretion was indirectly accounted for by subtracting the total activity associated with all the organs/tissues from the total activity administered in each animal [25].

### 2.11. Clinical Studies

Clinical studies involved a total of 10 administrations in patients suffering from metastatic castration-resistant prostate carcinoma. The patients had earlier undergone treatment with various drugs such as docetaxel, enzalutamide, cabazitaxel, and carboplatin prior to their recruitment for ^177^Lu-PSMA-617 therapy. All the patients recruited for the present study had progressive disease despite undergoing chemotherapy and androgen deprivation therapy. Additionally, all the patients had histopathologically and biochemically proven prostate carcinoma with significantly high levels of PSA (prostate-specific antigen). All the patients recruited for the clinical study had undergone pretherapy whole-body PET-CT scanning with ^18^F-PSMA-1007, in order to ascertain the suitability of PSMA radiotherapy. On average, 5.18 GBq (140 mCi) of the radiolabeled ^177^Lu-PSMA-617 preparation was administered in each patient and posttherapy scintigraphic images were recorded 24 h after the administration of ^177^Lu-PSMA-617. For a couple of patients, the second therapeutic dose of ^177^Lu-PSMA-617 was given after two months of administration of the first therapeutic dose and imaging was performed again at 24 h postadministration.

## 3. Results

### 3.1. Formulation of Freeze-Dried PSMA-617 Kits

A total of 60 freeze-dried PSMA-617 kits, each comprising 100 *μ*g PSMA-617, 25 mg gentisic acid, 74 mg HEPES, and 8.5 mg NaOH in the lyophilized form, were prepared in 3 batches during the present course of the study. The kit vials were stored at 2-8°C immediately after preparation and were taken out just before the formulation of the ^177^Lu-PSMA-617 patient dose or other experimentations. The kit vials were always allowed to attain room temperature before using for various studies or formulation of ^177^Lu-PSMA-617 patient doses.

### 3.2. Quality Control Studies

**Figure 1:**
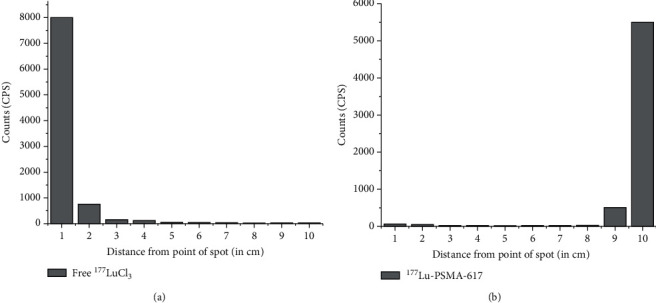
Paper chromatography patterns of (a) free ^177^LuCl_3_ and (b) ^177^Lu-PSMA-617 obtained using acetonitrile : water (1 : 1, *v*/*v*) as the eluting solvent.

**Figure 2:**
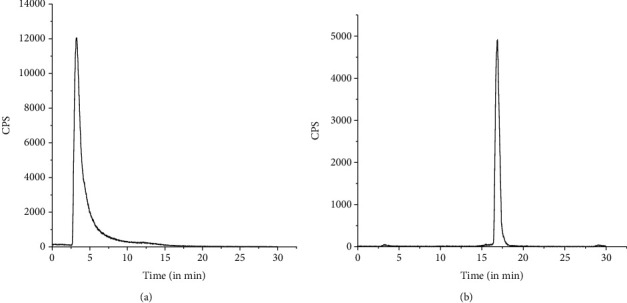
Typical HPLC profiles of (a) free ^177^LuCl_3_ and (b) ^177^Lu-PSMA-617.

Quality control studies were performed using PC as well as HPLC. In PC, carried out using acetonitrile : water (1 : 1 *v*/*v*) as the mobile phase, ^177^Lu-PSMA-617 moved towards the solvent front (*R*_f_ = 0.9-1.0), while uncomplexed ^177^LuCl_3_ remained at point of spotting (*R*_f_ = 0.0-0.1) under identical conditions. Analyses of PC data showed that the ^177^Lu-PSMA-617 complex could be obtained with >95% radiochemical purity using the freeze-dried PSMA-617 kit. Typical PC profiles of free ^177^LuCl_3_ and ^177^Lu-PSMA-617, recorded under identical conditions, are shown in Figures 1(a) and 1(b), respectively. The radiochemical purity of the ^177^Lu-PSMA-617 complex was further confirmed by HPLC studies, where ^177^Lu-PSMA-617 exhibited a retention time of 17.5 ± 1.0 min whereas free ^177^LuCl_3_ got eluted with a retention time of 3.5 ± 0.5 min. HPLC studies also showed that the ^177^Lu-PSMA-617 complex could be obtained with an average % radiochemical purity of 98.53 ± 0.38 using the freeze-dried PSMA-617 kit. A typical HPLC profile of uncomplexed ^177^LuCl_3_ is shown in Figure 2(a), while the same for the ^177^Lu-PSMA-617 complex, recorded under identical conditions, is shown in Figure 2(b).

### 3.3. Radiochemical Studies

Radiochemical studies carried out for determining the maximum activity which can be added in each kit vial revealed that maximum 7.7 *μ*g of Lu, which corresponds to the 2.5 : 1 ligand (PSMA-617) to metal (Lu) ratio, can be added without compromising the radiochemical purity of the ^177^Lu-PSMA-617 formulation. Considering that the specific activity of ^177^Lu available at our end is in the range of 740-925 MBq/*μ*g (20-25 mCi/*μ*g), maximum ^177^Lu activity that can be added in a kit vial will vary between 5.03 GBq (136 mCi) and 6.29 GBq (170 mCi). The addition of more than the above-mentioned activity range will reduce the radiochemical purity of the formulation below 95%. The effect of the addition of increasing concentration of carrier Lu on the percentage radiochemical purity of the ^177^Lu-PSMA-617 formulation is shown in Table 1.

Another set of radiochemical studies was performed to evaluate the maximum volume of ^177^LuCl_3_ that can be added in the kit vial without compromising the radiochemical purity of the ^177^Lu-PSMA-617 formulation. The results of these studies are shown in Table 2, which reveal that the resultant pH of reaction mixtures as well as radiochemical purity of ^177^Lu-PSMA-617 formulations remains almost unaltered due to the addition of up to 500 *μ*L of ^177^LuCl_3_ solution. As the addition of 500 *μ*L of ^177^LuCl_3_ is sufficient enough to prepare the patient dose of ^177^Lu-PSMA-617, studies with the addition of more than 500 *μ*L of ^177^LuCl_3_ were not attempted.

### 3.4. *In Vitro* Stability of ^177^Lu-PSMA-617 Formulated Using the Freeze-Dried Kit

**Figure 3:**
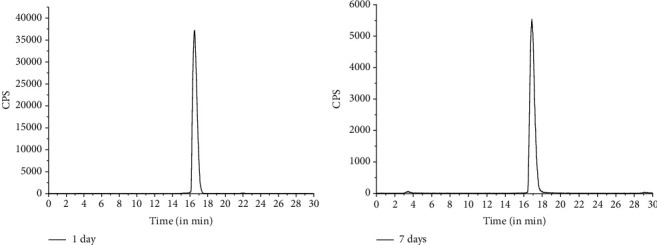
HPLC profiles depicting stability of ^177^Lu-PSMA-617, prepared using the freeze-dried PSMA-617 kit, at two different postpreparation time points viz. 1 d and 7 d.

*In vitro* stability studies revealed that the ^177^Lu-PSMA-617 complex prepared using the freeze-dried PSMA-617 is adequately stable till 7 d postpreparation at room temperature, up to which the studies were continued. The radiochemical purity of ^177^Lu-PSMA-617 remained >95% (96.74 ± 0.87) even after storing the formulation up to 7 d at room temperature. HPLC profiles of ^177^Lu-PSMA-617, recorded after 1 and 7 d of storage at room temperature, are shown in Figure 3.

### 3.5. Stability Studies in Human Serum

**Figure 4:**
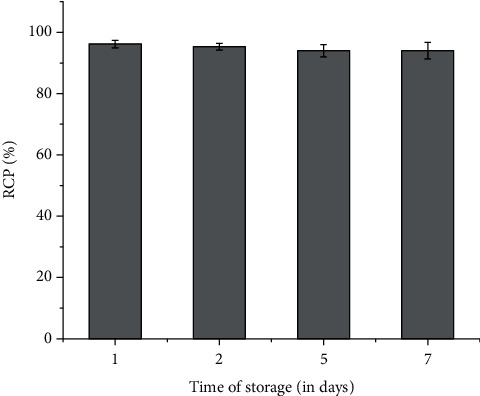
Stability of ^177^Lu-PSMA-617, prepared using the freeze-dried PSMA-617 kit, in human blood serum at different postpreparation time points viz. 1 d, 2 d, 5 d, and 7 d.

Stability studies, carried out in human blood serum, revealed that ^177^Lu-PSMA-617, formulated using the freeze-dried PSMA-617 kit, is adequately stable in the human blood serum as the formulation retained a radiochemical purity of 96.17 ± 1.21% after 1 d which reduced slightly to 94.81 ± 2.66% after 7 d of storage in the human serum at room temperature. The stability of the ^177^Lu-PSMA-617 formulation, prepared using the freeze-dried kit, in human blood serum at room temperature is shown in Figure 4.

### 3.6. Shelf Life of the Freeze-Dried PSMA-617 Kit

**Figure 5:**
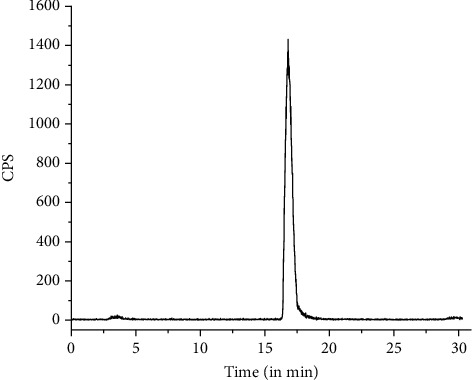
HPLC profile of ^177^Lu-PSMA-617, prepared using the freeze-dried PSMA-617 kit stored for one year at 2-8°C.

Shelf life determination of the freeze-dried kits was attempted in order to have an idea about the time period up to which the kits can be safely stored for the preparation of the ^177^Lu-PSMA-617 patient dose. Our studies revealed that the freeze-dried PSMA-617 kits could be used for the formulation of the ^177^Lu-PSMA-617 patient dose even after storage of one year at 2-8°C, as the radiolabeled agent could still be obtained with >95% radiochemical purity. A typical HPLC profile of the ^177^Lu-PSMA-617 complex, prepared using the freeze-dried PSMA-617 kit, stored for one year at 2-8°C is shown in Figure 5. The retention time and shape of the HPLC profile clearly indicate the unchanged nature of the ^177^Lu-PSMA-617 complex formulated using the PSMA-617 kit stored up to 1 year of preparation.

### 3.7. Biodistribution Studies

Results of the biodistribution studies, carried out in healthy male Wistar rats, were found to be almost identical with those reported in the literature [26] and are tabulated in Table 3. The study revealed rapid clearance of ^177^Lu-PSMA-617 from all the organs and fast excretion of non-accumulated activity through urinary route. The initially accumulated activity in some organs, such as GIT, kidneys, muscles, and skeletal components at 3 h postadministration also exhibited rapid clearance at longer time points (1 d, 2 d, and 7 d postadministration). An excretion of the order of 91.77 ± 2.96% IA and very low accumulation of activity in the liver (0.42 ± 0.02%  IA/organ) and gastrointestinal track (GIT) (1.37 ± 1.13%  IA/organ) at 3 h postadministration indicated that the biological distribution of ^177^Lu-PSMA-617 has remained unchanged when prepared using the freeze-dried kit [26].

### 3.8. Clinical Studies

**Figure 6:**
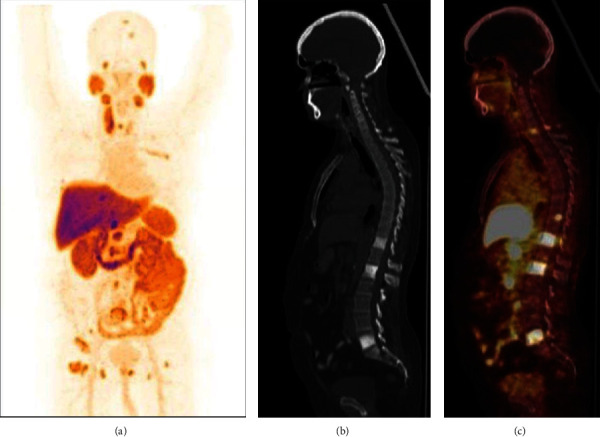
^18^F-PSMA-1007 (PET-CT) scans: (a) maximum intensity projection (MIP) PET image, (b) noncontrast CT (sagittal) image, and (c) PET-CT fused sagittal image showing multiple PSMA-avid lesions in bone (skull, spine, and right femur) and right-side cervical nodes.

**Figure 7:**
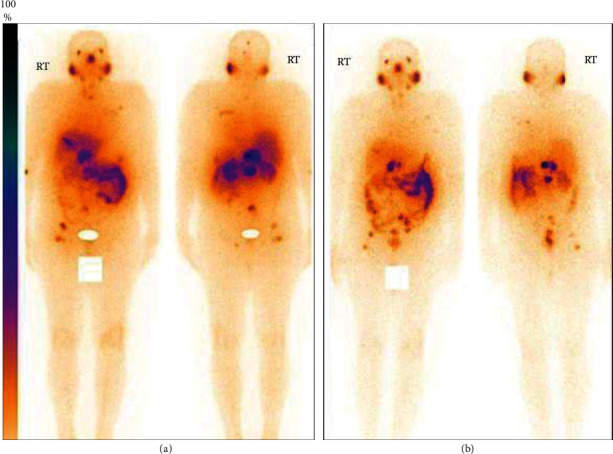
SPECT scans (anterior and posterior) of the same patient (a and b), obtained at 24 h postadministration of the first and second therapeutic doses of ^177^Lu-PSMA-617, prepared using the freeze-dried PSMA-617 kit, respectively.

A total of 10 patient doses of ^177^Lu-PSMA-617 were administered in patients suffering from metastatic castration-resistant prostate cancer. Whole-body scans, carried out at 24 h postadministration (first dose), revealed the uptake of ^177^Lu-PSMA-617, prepared using the in-house formulated freeze-dried PSMA-617 kits, in the cancerous lesions (including right-side cervical nodes and bone lesions (spine and right femur)), which were also picked up in the pretherapy PET-CT scans obtained using ^18^F-PSMA-1007 (Figures 6(a)–6(c) and 7(a)). However, an area of increased ^177^Lu-PSMA-617 uptake was also noted in the right lobe of the liver. Two months after the administration of the first dose of ^177^Lu-PSMA-617, a visual reduction in the number as well as size of the cancerous lesions (exhibiting shrinking of earlier documented right-side neck nodes, bones, and liver lesions) was observed while carrying out scintigraphic imaging after 24 hours of administration of the second therapeutic dose of ^177^Lu-PSMA-617 (Figure 7(b)) indicating the potential of the radiotherapeutic intervention.

## 4. Discussion

It is documented in the recent literature that prostate cancer is the most frequently diagnosed cancer among men in 105 out of 185 countries of the world, notably in the Americas, Northern and Western Europe, Australia/New Zealand, and much of sub-Saharan Africa. The disease is also the leading cause of cancer death among men in 46 countries, particularly in sub-Saharan Africa and the Caribbean [2]. As per the estimate of the American Cancer Society, about 1 man in 9 will be diagnosed with prostate cancer during his lifetime and 1 man in 41 will die of prostate cancer [29].

Detecting the prostate cancer at the early stage is the key to reduce the mortality rate, as catching the disease in its initial stage not only offers more treatment options but also increases the probability of survival to a great extent. Prostate cancer can often be found early by testing for prostate-specific antigen (PSA) levels in a man's blood or by digital rectal examination (DRE). However, these tests are not very accurate and often produce either false-positive or false-negative results. The recent success of molecular imaging techniques involving the use of radiolabeled PSMA ligands, such as ^68^Ga-PSMA-11 and ^18^F-PSMA-1007, in detecting the prostate cancer at the early stages of the disease has made this method quite popular for screening of prostate cancer in the recent times [4–9, 30, 31]. Consequently, therapeutic intervention of ca prostate utilizing the PSMA ligand labeled with suitable therapeutic radionuclide has gained immense popularity during the past few years. A cursory glance at the ongoing trials registered in the website ClinicalTrials.gov reveals that there are several phase I and phase II clinical trials presently underway with ^177^Lu-PSMA in advanced castration-resistant prostate cancer patients. Additionally, the first phase III study (termed as VISION) has also been initiated with an objective of investigating the safety and effectiveness of ^177^Lu-PSMA therapy against the current standard treatments for patients with progressive PSMA-positive, castration-resistant, postchemotherapy metastatic prostate cancer [32]. Therefore, the demand of ^177^Lu-PSMA-617 is expected to increase rapidly in the coming years, subject to the positive outcome of the ongoing clinical trials.

At present, hospital radiopharmacies at the healthcare facilities, situated at various countries across the globe, dealing with prostate cancer patients are engaged in preparing multiple doses of ^177^Lu-PSMA-617 using the wet chemistry protocol for providing radionuclide therapy to such patients. However, manufacturing of radiolabeled drugs through the in situ wet chemistry protocol comes with their own challenges, such as preparation of various solutions and buffers of proper strengths and maneuvering of right pH postaddition of multiple ingredients in the presence of high level of activity [22]. Additionally, availability of ^177^LuCl_3_ across different institutions in different radioactive concentration and specific activity further increases the challenges [33]. Formulation of freeze-dried kits where all the necessary ingredients are already present in a preoptimized manner eases the job of preparation of patient doses of therapeutic radiopharmaceuticals at the hospital radiopharmacy. By keeping this in mind, an attempt was made to formulate a freeze-dried kit of PSMA-617 which will enable convenient and single-step preparation of the ^177^Lu-PSMA-617 patient dose at the hospital radiopharmacy using ^177^Lu having a wide range of specific activity and radioactive concentration. Accordingly, a freeze-dried PSMA-617 kit was developed and used for the formulation of the patient dose of ^177^Lu-PSMA-617. Our present study showed that a single kit vial is sufficient for the preparation of 3.70 GBq (100 mCi) patient dose of ^177^Lu-PSMA-617, even when ^177^Lu having specific activity as low as 555 GBq/*μ*g (15 mCi/*μ*g) is used for the preparation.

^177^Lu-PSMA-617, prepared using the freeze-dried kit, showed adequate stability in human blood serum and a similar biodistribution pattern, reported for the same complex in literature. Clinical studies, although limited to only 10 administrations, showed excellent accumulation of the radiotracer in the cancerous lesions. The long shelf life of the kit can be considered another additional merit as such kits can be prepared anywhere and supplied all over the globe maintaining the adequate storage conditions.

A comparison of the present work with the similar studies reported in the contemporary literature revealed few important features of the present study (Table 4). Though the formulation of ^177^Lu-PSMA-617 has been reported widely [21, 34–37], comparison of the present work was primarily made only with those studies where emphasis was given on the radiochemical parameters for the formulation of patient doses of ^177^Lu-PSMA-617 [35–37]. While the majority of these studies described ready-to-use multidose formulation of ^177^Lu-PSMA-617 [35, 36], the present work describes formulation of a single patient dose of the agent using a freeze-dried kit. Therefore, a direct comparison of the present work with these studies cannot be made. Only Luna-Gutiérrez et al. documented the formulation of the ^177^Lu-PSMA-617 patient dose employing a freeze-dried kit [37]. However, the freeze-dried PSMA-617 kit reported by Luna-Gutiérrez et al. is meant for multidose formulation, and such type of kit is useful for the formulation of ^177^Lu-PSMA-617 only after pooling either 5 or 10 patients. Therefore, such kit may not be of much use when it has to be used for single or couple of patients owing to the economic reasons. However, the freeze-dried PSMA-617 kit described in the present study is for the formulation of a single patient dose and hence can be useful for both small and large radionuclide therapy centers. Another marked difference between the present study and that reported by Luna-Gutiérrez et al. [37] is the nature of ^177^Lu used for the formulation of ^177^Lu-PSMA-617, as the former uses comparatively low specific activity (n,*γ*) ^177^Lu, while the latter utilizes no-carrier-added ^177^Lu. Therefore, the freeze-dried PSMA-617 kit being reported in the present work will be useful even to those radionuclide therapy centers which do not have access to high specific activity no-carrier-added ^177^Lu.

## 5. Conclusions

Methodology of formulation of lyophilized PSMA-617 kits, suitable for the easy and convenient formulation of the ^177^Lu-PSMA-617 patient dose at the hospital radiopharmacy using the low/medium specific activity ^177^Lu, has been standardized. Radiochemical studies showed that the developed kits can be successfully used for the formulation of the ^177^Lu-PSMA-617 patient dose with high radiochemical purity even if lutetium-177 used for the preparation has low specific activity and/or low radioactive concentration. A long shelf life of the developed freeze-dried kits along with an adequate *in vitro* stability of the ^177^Lu-PSMA-617 formulation over a period of 7 days indicated the robust nature of the developed kit. Successful clinical translation subsequent to the pharmacokinetic study in a small animal model confirmed the usefulness of the developed PSMA-617 kit towards its use for the convenient, rapid, and single-step formulation of the ^177^Lu-PSMA-617 patient dose at the hospital radiopharmacy.

## Figures and Tables

**Table 1:** Variation of radiochemical purity of ^177^Lu-PSMA-617 with the addition of increasing amount of carrier Lu in the PSMA-617 kit vial.

Amount of radioactive Lu in *μ*g	Amount of nonradioactive Lu in *μ*g	Ligand to metal ratio^∗^ (*L* : *M*)	Percentage radiochemical purity
1 (20 mCi, 740 MBq)	3.2	4 : 1	98.97 ± 0.98
1 (20 mCi, 740 MBq)	4.6	3 : 1	98.25 ± 1.02
1 (20 mCi, 740 MBq)	5.7	2.5 : 1	97.65 ± 1.17
1 (20 mCi, 740 MBq)	7.4	2 : 1	91.82 ± 2.49

^∗^Amount of PSMA-617 in the kit vial is 100 *μ*g (95.9 nmol).

**Table 2:** Variation of radiochemical purity of ^177^Lu-PSMA-617 with the addition of increasing volume of ^177^LuCl_3_ (in 0.01 M HCl) in the kit vial.

Volume of ^177^LuCl_3_ (0.01 M HCl) added in the kit (in mL)	pH of the resulting solution	Percentage radiochemical purity
0.2	5.0	99.25 ± 0.66
0.3	4.0-5.0	98.75 ± 0.94
0.4	4.0-5.0	98.25 ± 1.36
0.5	4.0-5.0	97.88 ± 1.01

**Table 3:** Biodistribution pattern of ^177^Lu-PSMA-617, prepared using a freeze-dried PSMA-617 kit in normal male Wistar rats (*n* = 3).

Organ	% injected activity (%IA)/organ			
	3 h	1 d	2 d	7 d
Blood	0.27 ± 0.32	0.00 ± 0.00	0.00 ± 0.00	0.00 ± 0.00
Liver	0.42 ± 0.02	0.24 ± 0.04	0.15 ± 0.01	0.03 ± 0.02
GIT	1.37 ± 1.13	2.29 ± 0.51	1.55 ± 0.13	1.94 ± 0.54
Kidneys	3.47 ± 0.82	1.62 ± 0.38	1.08 ± 0.13	0.38 ± 0.05
Stomach	0.39 ± 0.16	0.07 ± 0.02	0.23 ± 0.23	0.08 ± 0.10
Heart	0.04 ± 0.04	0.00 ± 0.00	0.00 ± 0.00	0.00 ± 0.00
Lungs	0.06 ± 0.01	0.03 ± 0.01	0.03 ± 0.01	0.01 ± 0.01
Muscle	1.39 ± 0.81	0.80 ± 0.43	0.28 ± 0.22	0.51 ± 0.43
Skeleton	0.80 ± 0.23	0.43 ± 0.06	0.56 ± 0.06	1.22 ± 0.55
Spleen	0.02 ± 0.00	0.02 ± 0.01	0.01 ± 0.00	0.01 ± 0.00
Excretion#	91.77 ± 2.96	94.50 ± 1.66	96.11 ± 2.10	95.82 ± 1.53

^#^Percentage of activity excreted is determined by subtracting the activity accounted in all the organs from the total injected activity. Data are expressed in the form of average ± standard deviation derived from three replicate studies.

**Table 4:** Brief comparison of the present study with the similar reported studies.

Study	Kit-based formulation	^177^Lu	Multidose/single dose	Clinical efficacy
Chakraborty A. et al. [35]	No	Carrier added (0.66-0.81 GBq/mg)	Multidose (6 to 7)	Evaluated
Chakraborty S. et al. [36]	No	Carrier added (814-962 GBq/mg)	Multidose (5 to 8)	Not reported
Gutiérrez ML et al. [37]	Yes	Carrier free (40 GBq/mL)	Multidose (5 to 10)	Not reported
Present study	Yes	Carrier added (740-925 MBq/*μ*g)	Single dose	Evaluated

## Data Availability

All data relevant to the study are available in the manuscript.
